# Denture-associated biofilm infection in three-dimensional oral mucosal tissue models

**DOI:** 10.1099/jmm.0.000677

**Published:** 2018-01-11

**Authors:** Daniel J. Morse, Melanie J. Wilson, Xiaoqing Wei, Michael A. O. Lewis, David J. Bradshaw, Craig Murdoch, David W. Williams

**Affiliations:** ^1^​ Oral and Biomedical Sciences, School of Dentistry, Cardiff University, Cardiff, UK; ^2^​ GlaxoSmithKline Consumer Healthcare, Weybridge, Surrey, UK; ^3^​ School of Clinical Dentistry, University of Sheffield, Sheffield, UK

**Keywords:** biofilm, tissue model, candidosis, denture stomatitis, oral mucosa, infection

## Abstract

**Purpose:**

*In vitro* analyses of virulence, pathogenicity and associated host cell responses are important components in the study of biofilm infections. The *Candida*-related infection, denture-associated oral candidosis, affects up to 60 % of denture wearers and manifests as inflammation of palatal tissues contacting the denture-fitting surface. Commercially available three-dimensional tissue models can be used to study infection, but their use is limited for many academic research institutions, primarily because of the substantial purchase costs. The aim of this study was to develop and evaluate the use of *in vitro* tissue models to assess infections by biofilms on acrylic surfaces through tissue damage and *Candida albicans* virulence gene expression.

**Methodology:**

*In vitro* models were compared against commercially available tissue equivalents (keratinocyte-only, SkinEthic; full-thickness, MatTek Corporation). An *in vitro* keratinocyte-only tissue was produced using a cancer-derived cell line, TR146, and a full-thickness model incorporating primary fibroblasts and immortalised normal oral keratinocytes was also generated. The *in vitro* full-thickness tissues incorporated keratinocytes and fibroblasts, and have potential for future further development and analysis.

**Results:**

Following polymicrobial infection with biofilms on acrylic surfaces, both in-house developed models were shown to provide equivalent results to the SkinEthic and MatTek models in terms of tissue damage: a significant (*P*<0.05) increase in LDH activity for mixed species biofilms compared to uninfected control, and no significant difference (*P*>0.05) in the expression of most *C. albicans* virulence genes when comparing tissue models of the same type.

**Conclusion:**

Our results confirm the feasibility and suitability of using these alternative *in vitro* tissue models for such analyses.

## Introduction

Biofilms are highly significant in human infection, and these complex polymicrobial communities of micro-organisms can develop on biotic or abiotic surfaces [1, 2]. They develop according to a defined series of events, and are highly structured, with distinct microenvironments within the EPS supporting the growth of a diverse range of micro-organisms [2–7]. A key component of this is their inherent antimicrobial tolerance [8] and resistance to host defence molecules and mechanisms. Biofilms frequently occur on the surfaces of medical devices and are often implicated in healthcare-associated infections [9–14]. It is well documented that the oral cavity (with numerous different surfaces such as the surface of teeth, gingival tissue and mucosa) can support the growth of up to 1000 different species of micro-organisms, including bacteria [15–17] and fungi [18]. Dental plaque is a well-studied example of a polymicrobial biofilm in the oral cavity [19, 20] and is responsible for the most prevalent human infections, namely dental caries and periodontal disease.

Dental prostheses are common, particularly in the elderly population, and serve to maintain normal oral function and aesthetic appearance. Dentures are one such example, where, unlike living epithelia, this acrylic material lacks a natural sloughing capacity and, coupled with the inevitable formation of a salivary pellicle on the denture surface [21], serves to aid the establishment and retention of biofilms.

Chronic erythematous candidosis (*Candida*-associated denture stomatitis; DS) is a frequent oral infection that affects up to 60 % of denture wearers [22] and is associated with biofilms containing fungi of the genus *Candida*. DS presents as areas of inflammation on the palatal mucosa in close proximity to the denture acrylic, the extent of which is classified according to Newton’s classification [23]. The infection occurs as a result of poor oral and denture hygiene, along with a number of other predisposing factors, including tobacco use, diabetes and impaired immunocompetence [24–26].

*Candida* are generally regarded as the main causative agents of DS and current management strategies target the fungal component. However, it is becoming increasingly apparent that bacteria and *Candida* interact within biofilms [27], and the role of bacteria in the pathogenesis, and thus the prognosis and management, of DS warrants further evaluation.

Tissue models are valuable tools for *in vitro* analysis of the pathogenicity of biofilms and associated host cell responses. Several commercially available constructs have been used to undertake such investigations [27–30], and they are necessary to gain greater insight into the complex relationship between host and microbes, particularly in the context of biofilm infections.

Two types of keratinocyte-only oral mucosal epithelium tissue models are commercially available, namely SkinEthic Reconstituted Human Oral Epithelium (RHOE) (EpiSkin, Lyon, France) and EpiOral (MatTek Corporation, Ashland, MA, USA). In addition, there is a full-thickness oral mucosa model incorporating a fibroblast-populated *lamina propria* composed of collagen and overlaid with keratinocytes (EpiOral FT, MatTek Corporation).

Keratinocyte-only models are comparatively simplistic, containing the epithelial layer, but lack a collagen matrix and fibroblast cells. Thus, potential analyses are limited when compared to full-thickness tissue models. Furthermore, the commercially available models provided are ‘static’, i.e. they are ‘ready-to-use’ products, with no potential to incorporate additional cells, such as immune cells or endothelial cells, to make them more representative of normal tissues.

This study developed and evaluated two *in vitro* tissue models as alternatives to the commercially available constructs, and for the first time used them to assess the effects of infection with denture biofilms. Specifically, the biofilm pathogenicity and host cell responses toward DS infections were analysed.

## Methods

### Cell culture and conditions

TR146 keratinocytes (obtained from Cancer Research UK) were cultured in Dulbecco’s modified Eagle's medium (DMEM; 11965–092, Life Technologies) supplemented with 4.5 g glucose l^−1^, 10 % (v/v) foetal bovine serum (FBS), 2.5 mM l-glutamine, and 100 U penicillin ml^−1^ and 100 µg streptomycin ml^−1^ (Life Technologies). The cells were cultured and maintained in T75/T175 culture flasks in a humidified incubator at 37 °C with 5 % CO_2_, 95 % air.

Primary human oral fibroblasts isolated from biopsies obtained from the buccal and gingival oral mucosa from patients during routine dental procedures with written, informed consent [31] (ethical approval number 09/H1308/66) kindly provided by Dr Helen Colley, University of Sheffield, were cultured in DMEM supplemented with 4.5 g glucose l^−1^, 10 % (v/v) FBS, 2.5 mM l-glutamine, 100 U penicillin ml^−1^ and 100 µg streptomycin ml^−1^ in T175 flasks in a humidified incubator at 37 °C with 5 % CO_2_, 95 % air.

FNB6 keratinocytes (obtained from Dr Keith Hunter, University of Sheffield, MTA provided by Cancer Research UK) were cultured in DMEM supplemented with 4.5 g glucose l^−1^, 10 % (v/v) FBS, 2.5 mM l-glutamine, and 100 U penicillin ml^−1^ and 100 µg streptomycin ml^−1^ in T175 flasks in a humidified incubator at 37 °C with 5 % CO_2_, 95 % air. Gamma-irradiated mouse-3T3 fibroblast cells were co-cultured at a density of approximately 3×10^5^ cells per flask as a feeder layer.

### Isolation of type I rat tail collagen

Rat tail type I collagen was isolated from the tails of Wistar rats. Briefly, surgically removed rat tails were folded and twisted approximately 4–5 cm from the base to expose the collagen fibres. The fibres were removed, washed in sterile PBS and dissolved for 7 days in 0.1 M sterile acetic acid at 4 °C with continuous stirring. The collagen was freeze-dried, redissolved in 0.1 M acetic acid to a stock concentration of 5 mg collagen ml^−1^, and then stored at 4 °C.

### Keratinocyte-only tissue model

TR146 cells were washed with PBS, and 3 ml of 0.25 % (w/v) trypsin-EDTA solution (Life Technologies, UK) was added to the flasks. The flasks were then incubated for 5 min to detach cells. The cells were collected into sterile plastic universal containers, centrifuged to pellet at 1500 rev min^−1^ for 5 min, and then suspended at 1–2×10^6^ cells ml^−1^ in fresh keratinocyte DMEM culture medium [DMEM/F12 was supplemented with 4.5 g glucose l^−1^, 10 % (v/v) FBS, 2.5 mM l-glutamine, 10 ng epidermal growth factor ml^−1^, 0.5 µg insulin ml^−1^, 1.8×10^4^ U cholera toxin ml^−1^ and 5 µg adenine ml^−1^]. A 500 µl volume of the standardised cell suspension was added to each 12 mm diameter (0.4 µm pore size) polycarbonate cell culture insert (PIHP01250, Merck Millipore) and then 2 ml of fresh keratinocyte DMEM was added to the well. The cells were incubated to allow attachment for 48 h, after which the medium was removed from both the insert and the well, and 600 µl fresh keratinocyte DMEM was added to the well. The medium was replenished every 2 days to maintain growth, taking care not to disturb the tissue, but removing any excess medium from inside the insert if necessary.

### Co-culture tissue model

Full-thickness oral mucosal models were produced as previously described. [32, 33] Primary human oral fibroblasts were washed and detached form the culture flasks with 0.25 % trypsin-EDTA, centrifuged and suspended at 1×10^6^ cells ml^−1^ in DMEM. The collagen mixture was then prepared (sufficient for ten inserts), containing 1 ml of reconstitution buffer (22 mg sodium bicarbonate ml^−1^, 20 mM HEPES buffer, 0.062 M NaOH), 1 ml 10×DMEM (Sigma), 0.83 ml FBS, 0.1 ml l-glutamine and 6.73 ml collagen type 1 dissolved in 0.1 M acetic acid (5 mg collagen ml^−1^ extracted from rat tails), and kept on ice. The collagen mixture was gently but thoroughly mixed by hand, and the pH was adjusted by the addition of 1 M NaOH to be neutral. Fibroblasts were added into the collagen mixture (at a final concentration of 2.5×10^5^ cells per insert) and mixed gently until homogenous, and then 500 µl of the collagen and cell mixture was added to each insert. The plates were incubated at 37 °C in 5 % CO_2_, 95 % air for 30 min to allow the collagen to set, and then 2 ml of DMEM added to the well and the plates returned to the incubator. After 48 h incubation, cultured TR146 or FNB6 keratinocytes were detached with 0.25 % trypsin-EDTA and suspended to 1×10^6^ cells ml^−1^ in DMEM, before 500 µl of the cell suspension was added to each insert on top of the collagen and fibroblasts. These cells were cultured for a further 48 h and then brought to the air–liquid interface (ALI). Culture medium was removed from the inside of the insert and the well, and 600 µl of fresh keratinocyte medium [DMEM, Hams F12, supplemented with 10 % (v/v) FBS, 100 U penicillin ml^−1^, 100 µg streptomycin ml^−1^, 0.625 µg amphotericin B ml^−1^, 0.025 µg adenine ml^−1^, 1.36 ng 3,3,5-tri-iodothymine ml^−1^, 5 µg apo-transferrin ml^−1^, 4 µg hydrocortisone ml^−1^, 5 ng epidermal growth factor ml^−1^, 8.47 ng cholera toxin ml^−1^ and 5 µg insulin ml^−1^) was added to the well, leaving the cells in the insert at the ALI. These models were cultured for a further 14 days, with the medium being replenished every 2 days. Two days prior to the tissue model infection, the growth medium was adjusted to exclude the use of penicillin/streptomycin.

### Receipt and processing of commercial mucosal tissue models

Both the EpiSkin and MatTek Corporation tissue models were transported with the models set in ‘agar-like’ culture medium to restrict movement during transport, and upon receipt they were placed into fresh 12-well plates with fresh maintenance medium as provided by the manufacturer. The models were incubated in 5 % CO_2_ in a humidified incubator for at least 4 h prior to infection.

### Biofilm infection of tissue models

Circular acrylic [poly-(methyl methacrylate); PMMA] coupons (approximately 10 mm diameter, 2 mm thickness) were prepared and sterilised. These coupons were then preconditioned overnight with an artificial saliva solution [2.5 g porcine stomach mucin l^−1^ (Sigma), 0.35 g sodium chloride l^−1^, 0.2 g potassium chloride l^−1^, 0.2 g calcium chloride dehydrate l^−1^, 2 g yeast extract l^−1^, 1 g Lab-Lemco powder l^−1^ (Sigma), 5 g proteose peptone l^−1^ (Sigma) and 1.25 ml 40 % (w/v) urea solution l^−1^]. The biofilms were prepared on the acrylic coupons as detailed previously [27]. Briefly, the biofilms were grown on PMMA coupons and contained the following micro-organisms: single-species *Candida albicans* ATCC 90028, bacteria-only (*Streptococcus sanguinis* ATCC 10556, *S. gordonii* ATCC 10558, *Actinomyces viscosus* ATCC 15987 and *A. odontolyticus* NCTC 9935) and mixed species (*C. albicans* plus oral bacteria). The biofilms were prepared by the inoculation of 100 µl of each micro-organism suspension from a standardised concentration (as measured by optical density at 600 nm; a density of 1.0 for *C. albicans* and densities of 0.08–0.10 for bacterial cultures) for 2 h. Non-adhered cells were then removed and fresh medium was added to the wells. The biofilms were cultured for 72 h with daily medium change.

Immediately prior to tissue infection, the tissue culture medium was replaced with fresh culture medium, and the acrylic coupons with and without biofilms were inverted and placed in direct contact with the tissue models and incubated at 37 °C in 5 % CO_2_, 95 % air for 12 h. Post-infection, the acrylic coupons were carefully removed with forceps, and the tissue models were cut out of the insert with a scalpel and bisected. One section was used for histopathological processing and imaging, and the other was added immediately to 350 µl buffer RLT (Qiagen) with 1 % (v/v) β-mercaptoethanol and 500 µl phenol:chloroform:isoamyl alcohol (25 : 24 : 1, Sigma) and kept on ice prior to RNA extraction.

### Histopathological preparation and analysis of tissue models

Tissue models were fixed in 10 % (v/v) formal saline and then subjected to dehydration and formalin/paraffin fixation in a pathology cassette using Leica ASP300S processor. The samples were sectioned at 5 µm thickness and stained with haematoxylin and eosin using an automated staining machine (Thermo GLX Linistainer, Thermo Scientific).

### RNA extraction, purification and synthesis of cDNA

RNA was extracted using an RNeasy Mini-prep kit (Qiagen) and reverse-transcribed to cDNA using a reverse-transcription kit (Primer Design, UK) according to manufacturer’s instructions, as described by Cavalcanti *et al.* [27].

### qPCR analysis of tissue model infections

Real-time qPCR was performed as described previously [27]. The targeted putative virulence genes of *C. albicans* were *ALS1*, *ALS3* (agglutinin-like sequences), *EPA1* (epithelial adhesin), *SAP4* and *SAP6* (secreted aspartyl proteinases), *HWP1* (hyphal wall protein) and *PLD1* (phospholipase D), the primer sequences of which are detailed in Table 1. Human interleukin-18 (IL-18) was also evaluated for keratinocyte-only tissue model infections as described previously [27]. The primer sequences for the human genes are also detailed in Table 1. Analysis of relative gene expression was performed according to the ^ΔΔ^Ct method [34].

**Table 1.** *Candida* and human gene primer sequences used in qPCR analysis

**Target gene**	Sequence (5′ → 3′)
**ACT1** – housekeeping gene	FW – TGCTGAACGTATGCAAAAGG RV – TGAACAATGGATGGACCAGA
**ALS1** – agglutinin-like sequence	FW – CCCAACTTGGAATGCTGTTT RV – TTTCAAAGCGTCGTTCACAG
**ALS3** – agglutinin-like sequence	FW – CTGGACCACCAGGAAACACT RV – GGTGGAGCGGTGACAGTAGT
**EPA1** – epithelial adhesin	FW – ATGTGGCTCTGGGTTTTACG RV – TGGTCCGTATGGGCTAGGTA
**HWP1** – hyphae wall protein	FW – TCTACTGCTCCAGCCACTGA RV – CCAGCAGGAATTGTTTCCAT
**PLD1** – phospholipase D	FW - GCCAAGAGAGCAAGGGTTAGCA RV – CGGATTCGTCATCCATTTCTCC
**SAP4** – secreted aspartyl proteinase	FW – GTCAATGTCAACGCTGGTGTCC RV – ATTCCGAAGCAGGAACGGTGTCC
**SAP6** – secreted aspartyl proteinase	FW – AAAATGGCGTGGTGACAGAGGT RV – CGTTGGCTTGGAAACCAATACC
**β-actin** – human housekeeping gene	F – GAGCACAGAGCCTCGCCTTTGCCGAT R – ATCCTTCTGACCCATGCCCACCATCACG
**IL-18** – human interleukin 18 (pro-inflammatory cytokine)	F – CCTTCCAGATCGCTTCCTCTCGCAACAA R – CAAGCTTGCCAAAGTAATCTGATTCCAGGT

### Monocyte cell responses to bacterial lipopolysaccharide and heat killed *C. albicans*

THP-1 monocytes were cultured in RPMI 1640 medium (Life Technologies), supplemented with 10 % (v/v) FBS and 2 mM l-glutamine. The cells were seeded at a density of 2×10^5^ cells ml^−1^ in six-well plates prior to lipopolysaccharide (LPS) or heat-killed *C. albicans* (HKC) challenge. *C. albicans* cells were cultured overnight in yeast nitrogen base supplemented with 100 mM glucose as previously described [27], and heated in a water bath at 95 °C for 10 min. Heat-killing efficacy was confirmed by negative agar culture on Sabouraud dextrose agar. Bacterial LPS (isolated from *Escherichia coli*, Sigma) was prepared at a stock concentration of 1 mg ml^−1^ in PBS and diluted in RPMI 1640 (Life Technologies), supplemented with 10 % (v/v) FBS and 2 mM l-glutamine. THP-1 cells were cultured overnight in the presence or absence of 100 ng LPS ml^−1^, and then challenged overnight with HKC at varying densities. The cell culture conditioned medium was collected and stored at −80 °C.

### Quantification of secreted IL-23 cytokine protein by enzyme linked immunosorbent assay (ELISA)

IL-23 ELISA was performed on cell culture-conditioned medium using the Ready-Set-Go kit (eBioscience, Thermo Fisher Scientific) according to the manufacturer’s instructions. The resulting concentration of IL-23 in the conditioned medium was determined by plotting sample absorbance against the standard curve.

### Lactate dehydrogenase (LDH) activity of infected tissue models

Supernatant (*n*=3 for each group) was collected for the analysis of cell damage via lactate dehydrogenase activity assay (Pierce LDH cytotoxicity assay; Fisher Scientific, Cramlington, UK) according to the manufacturer’s instructions, and the results were normalised to acrylic-only (no biofilm) controls.

### Statistical analysis

Statistical analysis was performed with Prism version 6.0c (GraphPad Software, La Jolla, CA, USA). One-way ANOVA followed by Tukey’s multiple comparisons test was used to evaluate statistical differences between samples at 95 % confidence.

## Results

### *In vitro* tissue model culture

**Fig. 1.**
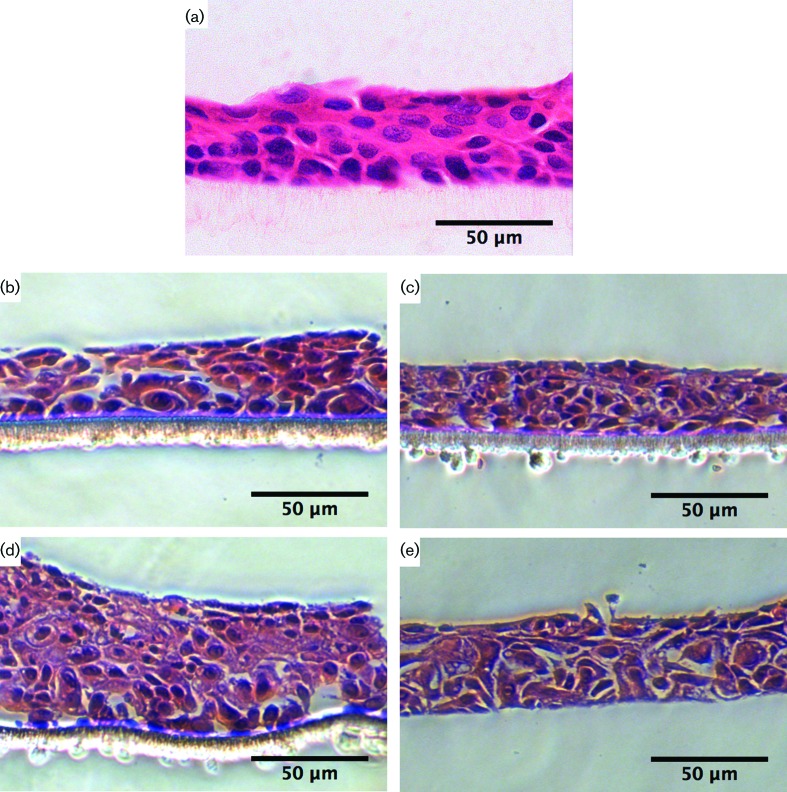
Representative light microscopy images of the *in vitro* keratinocyte-only tissue model, sectioned at 5 µm thickness. (a) SkinEthic reconstituted human oral epithelia (RHOE) tissue; (b) *in vitro* keratinocyte-only tissue seeded with 5×10^5^ cells cultured for 5 days; (c) *in vitro* keratinocyte-only tissue seeded with 5×10^5^ cells cultured for 10 days; (d) *in vitro* keratinocyte-only tissue seeded with 1×10^6^ cells cultured for 5 days; (e) *in vitro* keratinocyte-only tissue seeded with 1×10^6^ cells cultured for 10 days. Stained with haematoxylin and eosin. The scale bar represents 50 µm.

The development of an *in vitro* keratinocyte-only tissue model was achieved. This involved using different cell seeding densities and incubation periods, and it was compared to the SkinEthic RHOE model for tissue depth and structure (Fig. 1a). The cell densities, namely 5×10^5^ (Fig. 1b, c), and 1×10^6^ (Fig. 1d, e) cells per insert, and different incubation periods, including 5 days (Fig. 1b, d), and 10 days (Fig. 1c, e), showed varied tissue depths, but the observed cell morphology was typical of viable keratinocytes at all densities and incubation periods. The tissue depth was consistent across the supporting membrane for each tissue section, with the presence of tight cell-to-cell junctions being evident. Reduced tissue thickness was apparent at the lower seeding densities [5×10^5^ cells per insert (Fig. 1b, c) compared with 1×10^6^ cells (Fig. 1d, e)] per insert, but the thickness was similar to that of SkinEthic RHOE.

Growth at 5 days (following seeding with 1×10^6^ cells) resulted in a tissue thickness that was greater than that observed in the SkinEthic RHOE tissues. There were small differences in tissue thickness across the membrane, but no differences in cellular morphology for 5- or 10-day cultures. All of the models showed good stratification and limited cell differentiation.

**Fig. 2.**
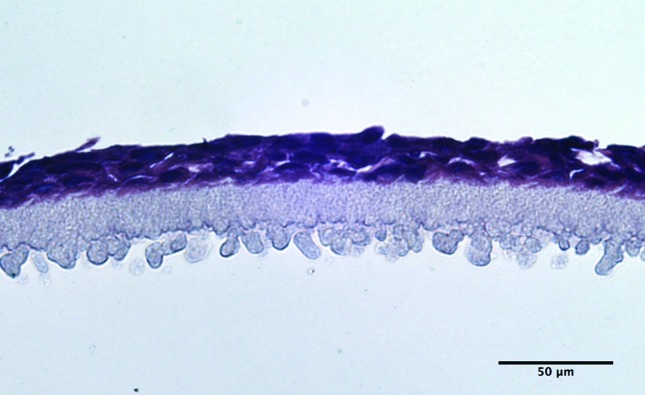
Light microscopy image showing a very thin epithelium when culturing is performed with medium covering the cells (e.g. not at the air–liquid interface). Stained with haematoxylin and eosin. The scale bar represents 50 µm.

During the development of the method, the importance of the ALI was noted. When the models were cultured submersed in culture medium, the thickness of the tissue was greatly reduced (Fig. 2) compared to those cultured at the ALI, and thus all of the tissue models were then cultured at the ALI to ensure good stratification of the epithelium. Culturing at the ALI is also known to promote keratinisation of the epithelium, which is present in normal oral mucosa, whereas when the culturing is submersed, keratinisation is restricted.

**Fig. 3.**
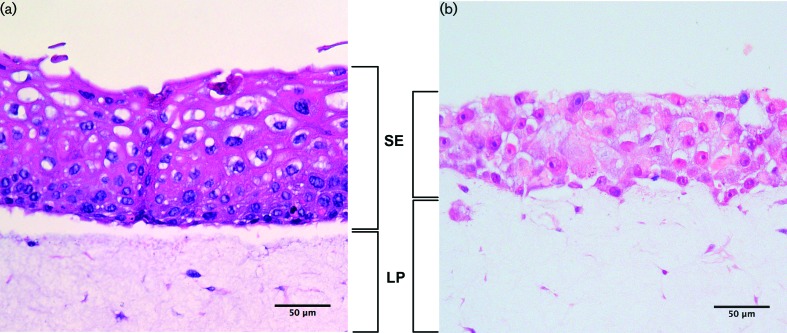
(a) *In vitro* full-thickness mucosal tissue using primary oral cells. Clear distinct layers of fibroblast-populated *lamina propria* (LP) in collagen, with good stratification and differentiation of the epithelia (SE). (b) *In vitro* full-thickness mucosal tissue using primary fibroblasts, with TR146 cancer-derived keratinocytes. Note the substantial lack of cellular maturation and organisation, and the reduced epithelial stratification compared with tissues developed using immortalised normal oral keratinocytes. Stained with haematoxylin and eosin. The scale bar represents 50 µm.

The development of the *in vitro* full-thickness tissue model development was more complex than that for the keratinocyte-only models. The required growth period was significantly longer: approximately 2 weeks, compared to just 5 days for the keratinocyte-only models. The longer culture period was necessary to achieve maturation of the cells and the establishment of a stratified epithelium (SE; Fig. 3a). As observed with the keratinocyte-only tissue models, consistent epithelial thickness was again evident across the membrane. Fig. 3(a) demonstrates a thick *lamina propria* (LP), with fibroblasts populating the collagen matrix relatively sparsely. Additionally, epithelial cell differentiation was observed within the SE layer, with several distinct keratinocyte morphologies visible. Also present was a basement membrane (a darker, more densely packed area of epithelium), with layers of stratification towards the upper region, where some keratinisation was evident.

When the cancer cell line TR146 was used in place of immortalised normal oral keratinocytes, the results were somewhat different (Fig. 3b) to those for immortalised normal oral keratinocytes (Fig. 3a). Specifically, whilst a number of layers of keratinocytes were visible in the epithelium, their organisation was substantially different. There was a reduction in the overall thickness of the epithelium, a lack of distinctly defined stratified layers, with no distinct basement membrane cells, and little cellular differentiation. The cellular morphology also appeared to be less representative of normal oral mucosa compared with models developed using immortalised normal oral keratinocytes. Furthermore, no keratinisation of the epithelium was evident, as was also observed with keratinocyte-only tissue models, which were developed using the same cell line.

### Denture-associated biofilm infection of keratinocyte-only tissues

**Fig. 4.**
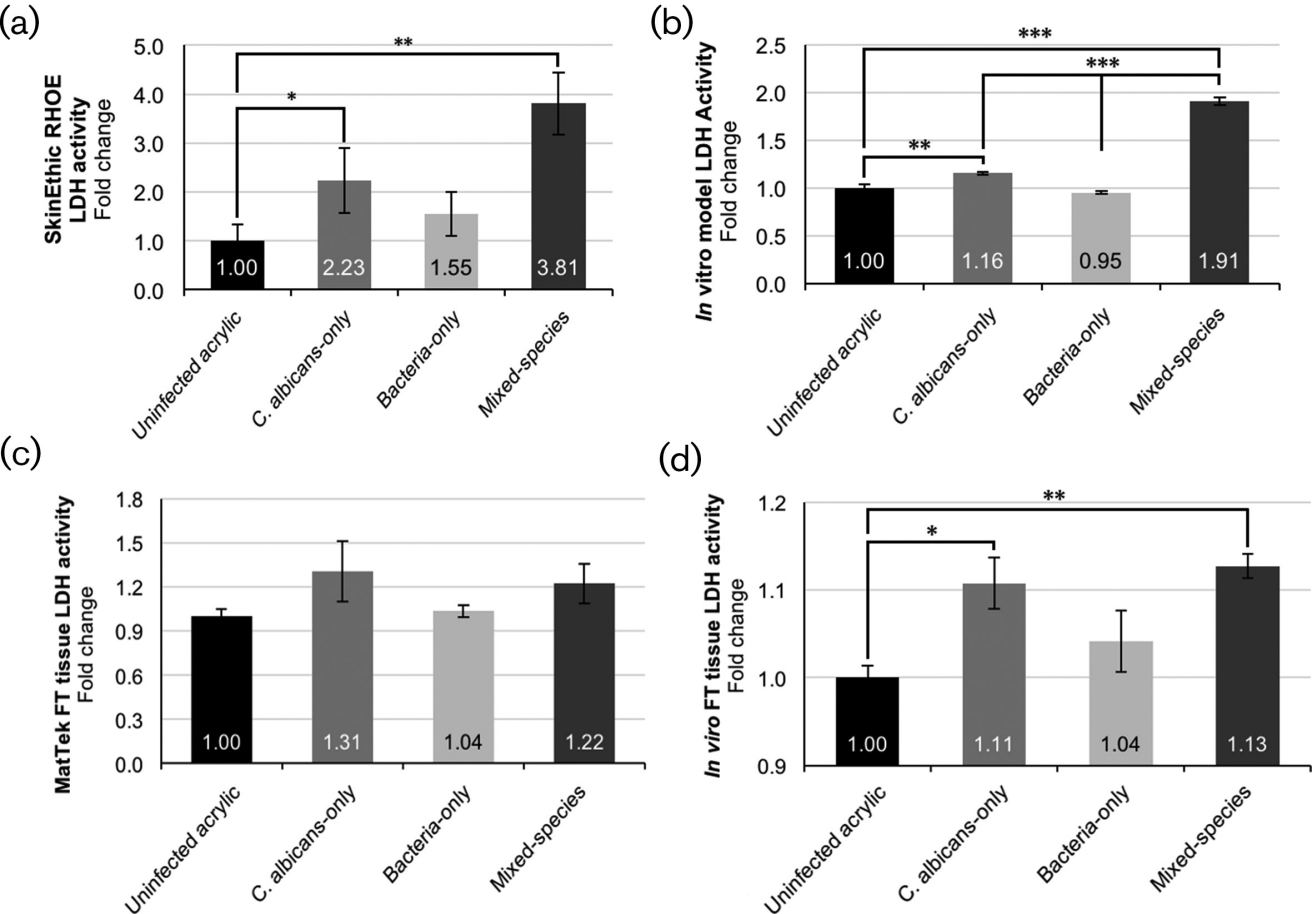
Biofilm-induced tissue damage as measured by the lactate dehydrogenase activity assay of infections. The results are expressed as fold change, normalised against an acrylic-only (no biofilm) control. (a) SkinEthic RHOE, (b) *in vitro* keratinocyte-only tissue, (c) MatTek EpiOral full-thickness tissue and (d) *in vitro* full-thickness tissue. All of the tissues show a similar pattern of damage; the largest increase in tissue damage is induced by mixed-species biofilms, compared to single-species and uninfected control acrylic coupons.

Infection of the SkinEthic RHOE tissues with mature biofilms developed on denture acrylic resulted in the same pattern of tissue damage as previously reported [27]. The tissue damage, as measured by lactate dehydrogenase (LDH) activity, showed that bacteria-only biofilm infections induced an increase in damage compared with acrylic coupons with no biofilms, although this was not statistically significant (*P*>0.05). However, a significant (*P*<0.05) increase occurred with *C. albicans*-only biofilms, and a further significant (*P*<0.01) increase in tissue damage was observed with mixed-species (bacteria and *C. albicans*) biofilms (Fig. 4a).

Biofilm infection of the *in vitro* keratinocyte-only models (Fig. 4b) resulted in a similar pattern of damage to that observed with infections of the SkinEthic RHOE. A significant (*P*<0.01) increase in tissue damage was caused by *C. albicans*-only biofilms relative to the acrylic coupons with no biofilms, and a further significant (*P*<0.001) increase was observed with mixed-species biofilm infections relative to both the *C. albicans*-only biofilms and bacteria-only biofilms, and the uninfected acrylic coupons.

### Denture-associated biofilm infection of full-thickness tissue models

A similar pattern of biofilm-induced tissue damage to the keratinocyte-only tissue models was evident with the MatTek EpiOral full-thickness tissue models; an increase in the damage caused by *C. albicans*-only and mixed-species biofilms, but no clear difference with bacteria-only biofilms. These differences, when compared with the uninfected acrylic coupons only, were not statistically significant (*P*>0.05) (Fig. 4c). However, *in vitro* full-thickness tissue model infections (Fig. 4d) once again showed a similar pattern of tissue damage, but with a significant (*P*<0.05) increase in LDH activity caused by *C. albicans*-only biofilm infections, and a similar, but even more significant (*P*<0.01), increase in mixed-species biofilms. Furthermore, as previously observed, no significant (*P*>0.05) differences in tissue damage were observed with infection using bacteria-only biofilms.

### *C. albicans* gene expression profile during biofilm infection of tissue models

The relative levels of expression of putative *C. albicans* virulence genes were compared to those of the housekeeping control gene, *ACT1*, and relative to a *C. albicans*-only biofilm control infection. The expression of all *C. albicans* virulence genes, with the exception of *ALS1,* followed the same pattern when compared between mixed-species biofilm infection of tissues of the same type, e.g. SkinEthic RHOE versus *in vitro* keratinocyte-only, and MatTek EpiOral full-thickness versus *in vitro* full-thickness tissues. Some variation in the extent of gene expression was evident, which highlights the innate variability of biofilms.

In order to establish whether there were differences between the commercial and in-house tissues with regard to the infecting biofilms, comparisons were again made of tissue models of the same type; namely SkinEthic RHOE versus *in vitro* keratinocyte-only tissues, and MatTek EpiOral versus *in vitro* full-thickness tissues. Overall, no significant differences in *C. albicans* gene expression were observed between infections of tissues of the same type for *ALS3, EPA1, HWP1, PLD1, SAP4* or *SAP6* genes. A significant (*P*<0.01) increase in the expression of *ALS1* was observed in *in vitro* full-thickness tissues compared with MatTek EpiOral, and a larger increase in the expression of *SAP4* was also observed in biofilms used to infect the *in vitro* full-thickness tissue compared with MatTek EpiOral tissues, but this difference was not statistically significant.

**Fig. 5.**
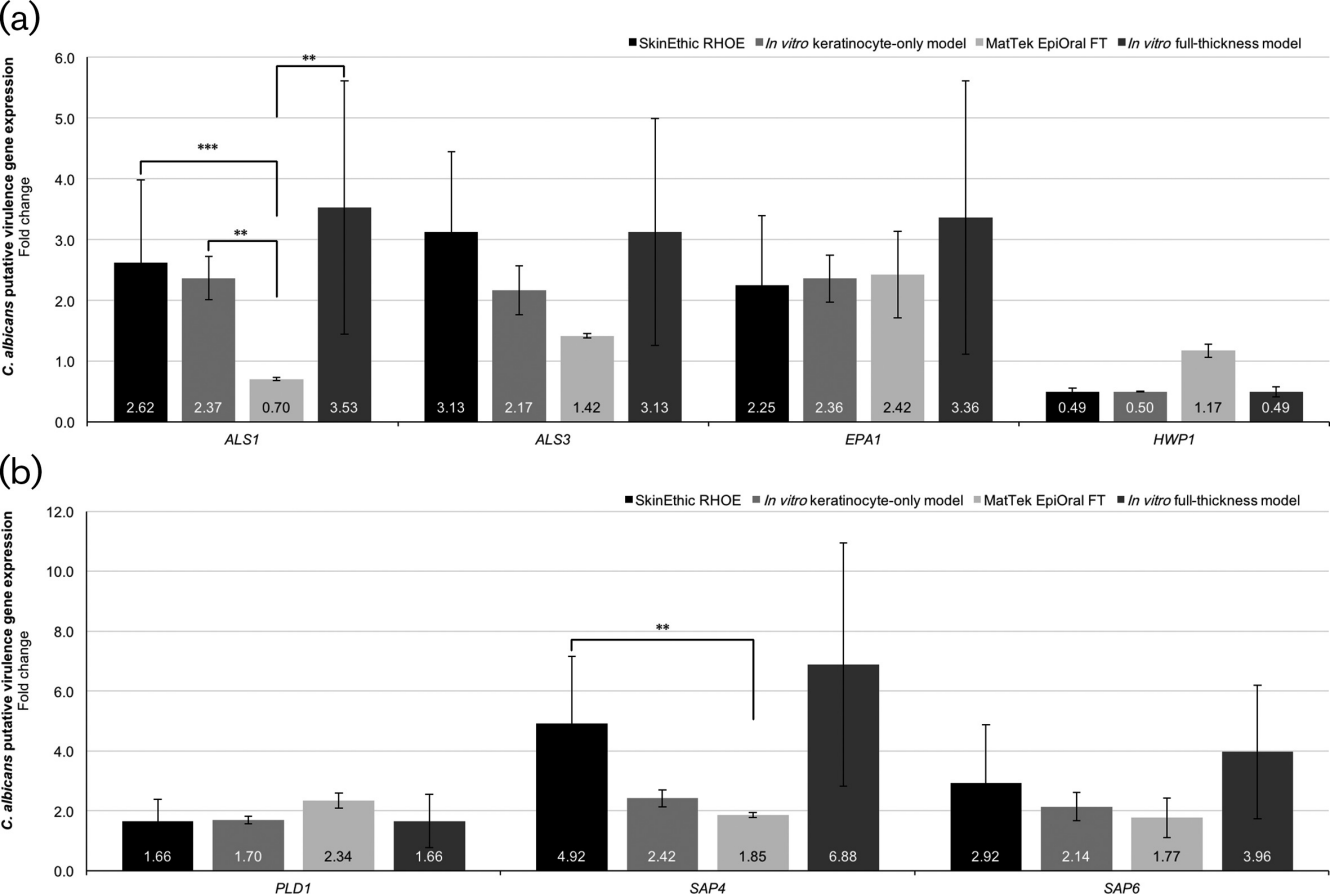
Expression of putative *Candida albicans* virulence genes of mixed-species biofilms post-tissue infection. The results are expressed as the fold change of samples relative to the housekeeping gene ACT1 against a normalised *C. albicans*-only biofilm control. No significant differences in *C albicans* gene expression were observed between tissue models of the same type (e.g. SkinEthic RHOE versus *in vitro* keratinocyte only, or MatTek EpiOral full-thickness versus *in vitro* full-thickness tissues) with the exception of full-thickness tissues for the *ALS1* gene.

The expression of the genes involved in adhesion, biofilm initiation and maturation, and formation of *C. albicans* hyphae (Fig. 5a; agglutinin-like sequences, *ALS1*, *ALS3*; epithelial adhesin 1, *EPA1*) was increased in mixed-species biofilms relative to *C. albicans*-only biofilms, and showed little difference between the different tissue infections, with the exception of *ALS1* in the MatTek EpiOral infections, where a decrease in expression was observed.

The *C. albicans* genes involved in pathogenicity by cell invasion and damage (Fig. 5b; phospholipase D1, *PLD1*; secreted aspartyl proteases, *SAP4*, *SAP6*) were also increased in the mixed-species biofilms for each of the tissue models. A significant increase in the gene expression of *SAP4* was observed in the SkinEthic RHOE tissue infections compared with MatTek EpiOral tissue infections, but no significant differences were observed between tissue models of the same type. Additionally, although there was no apparent increase in the expression of hyphal wall protein 1 (Fig. 5a; *HWP1*) expression during the infections in this study, we have previously shown that a significant proportion of hyphae are present relative to yeast cells [27] in these biofilms, and there was a substantial presence of hyphae in the microscopy of the tissue infection histological sections.

### Histological analyses of full thickness tissue model infections

The ability to perform histological analysis proved useful for determining the extent of infection, particularly with regard to damage to the epithelium, as previously evaluated with the RHOE keratinocyte-only model [27]. This study therefore focused on the evaluation of full-thickness tissue infections for histological analysis.

**Fig. 6.**
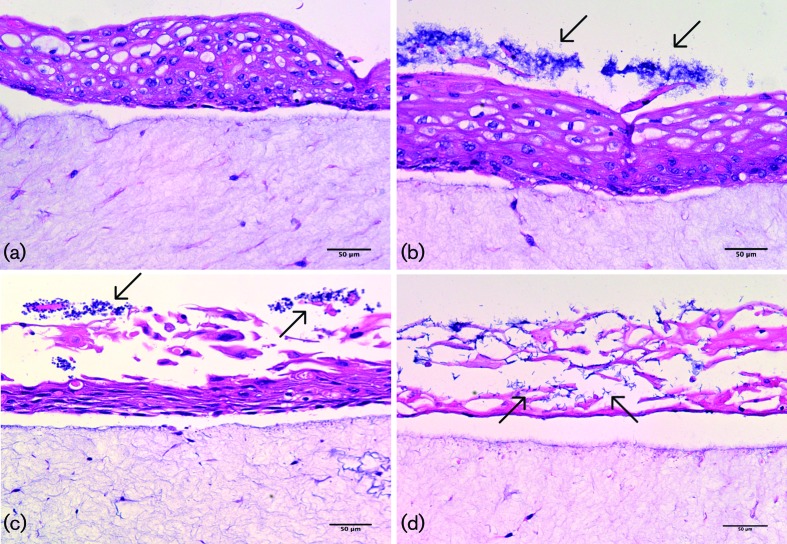
Typical light microscopy images of (a) full-thickness tissue after infection with acrylic coupons only (no biofilm), showing no damage to epithelium; (b) full-thickness tissue after infection with bacteria-only biofilm, showing slight tissue damage and clustering of biofilms on the epithelial surface (indicated by arrows); (c) full-thickness tissue after infection with *Candida albicans*-only biofilm, showing substantial epithelial damage and biofilms clustered on the surface of the epithelium (indicated by arrows); (d) full-thickness tissue after infection with mixed-species biofilm, showing extensive epithelial damage and microbial invasion through the epithelium (indicated by arrows). Stained with haematoxylin and eosin. The scale bar represents 50 µm.

**Fig. 7.**
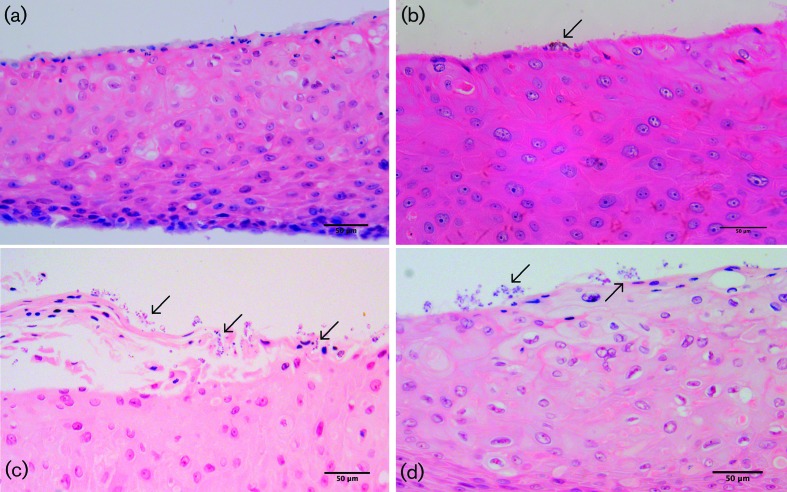
Typical light microscopy images of (a) MatTek full-thickness tissue after infection with acrylic coupons only (no biofilm), showing no damage to epithelium; (b) MatTek full-thickness tissue after infection with bacteria-only biofilm, showing very slight tissue damage and clustering of biofilms on the epithelial surface (indicated by arrows); (c) MatTek full-thickness tissue after infection with *Candida albicans*-only biofilm, showing slight epithelial damage and biofilms clustered on the surface of the epithelium (indicated by arrows); (d) MatTek full-thickness tissue after infection with mixed-species biofilm, showing slight epithelial damage and biofilms clustered on the surface of the epithelium (indicated by arrows). Stained with haematoxylin and eosin. The scale bar represents 50 µm.

**Fig. 8.**
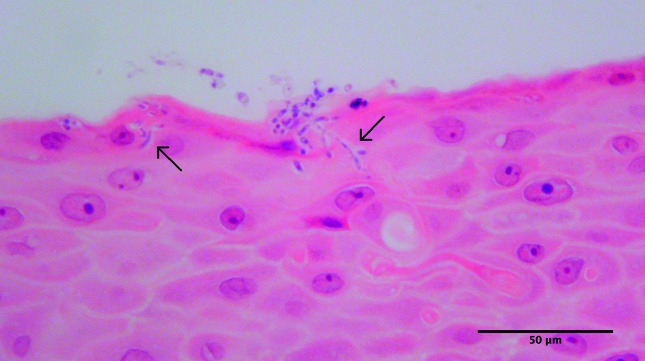
Representative light microscope image of the mixed-species biofilm infection of MatTek full-thickness tissues, demonstrating tissue damage caused by invading *Candida albicans* and bacteria as a result of biofilm infection. Stained with haematoxylin and eosin. The scale bar represents 50 µm.

The infections of *in vitro* full-thickness tissues (Fig. 6) and MatTek EpiOral tissues (Figs 7 and 8) showed some variation in the extent of the visible tissue damage caused by the infecting biofilms. This correlated with the variable LDH activity results previously described (Fig. 4c, d). Fig. 6(a) shows little to no damage caused by the acrylic coupon itself on the *in vitro* full-thickness tissues, as was also observed with the MatTek EpiOral full-thickness tissues (Fig. 7a), and similarly with bacteria-only biofilms in both tissue models (Figs 6b, and 7b). *C. albicans*-only biofilms caused substantial damage to *in vitro* full-thickness tissues (Fig. 6c) and some damage in MatTek EpiOral tissues (Fig. 7c), although to a lesser extent than *in vitro* tissues. Mixed-species biofilms caused substantial damage to the *in vitro* tissues, where there was significant penetration and epithelial invasion of *C. albicans* hyphae and bacteria. Some damage was observed in the MatTek EpiOral tissues when they were analysed histologically (Fig. 7d), which correlates with the LDH analysis, as previously discussed, and although the penetration and invasion of *C. albicans* hyphae was evident (Fig. 8), it was to a lesser extent than was observed in *in vitro* full-thickness tissues.

Information on the data underpinning the results presented here, including how to access them, can be found in the Cardiff University data catalogue at http://doi.org/10.17035/d.2017.0044151363.

## Discussion

### Three-dimensional tissue model development, limitations and advantages

Oral biofilms are of considerable clinical importance, and the ability to research the microbial interactions that underpin biofilm biology *in vitro* is important to understand and combat these infections. When *in vitro* tissue models provide valid and reproducible results, they are preferable, as they may remove the need for animal experimentation, a desirable characteristic for many funding bodies.

Commercially available tissue constructs have been used extensively to investigate infections [27–30, 35, 36]. Cavalcanti *et al.* [27] used such constructs to study denture biofilm infection using SkinEthic RHOE. This study is, however, the first to evaluate the use of both keratinocyte-only and full-thickness *in vitro* tissue models for denture biofilm infections, with a specific emphasis on biofilm-induced tissue damage in order to evaluate the *in vitro* tissues as an alternative to the commercially available constructs. The primary aim was to evaluate them in terms of a more cost-effective way of investigating biofilm infections of tissues, particularly in this study, related to infections associated with the oral mucosa.

The detection of secreted molecules in response to infection is dependent on the type and duration of the infection, as some cytokines typically take longer to accumulate in the supernatant to a level sufficient for detection with many assays, and therefore it takes longer to determine true differences between infections [35]. The biofilms used in this study were considered to be too pathogenic for sustained cytokine production to be allowed to proceed to a level that would be detectable in an *in vitro* assay without complete tissue destruction, but visual representation of tissue damage and microbial invasion was clearly evident.

### Biofilm infection varies between tissue types

This study aimed to compare commercially available and *in vitro* tissue models, both keratinocyte-only and full thickness, where we observed good similarity between the tissue models of the same type. The pattern of LDH activity was consistent between keratinocyte-only tissue models, as was *C. albicans* virulence gene expression. Additionally, the measurement of the relative expression of the *IL-18* gene in single versus mixed-species biofilms via RT-qPCR was also consistent between these models (Fig. S1, available in the online version of this article).

Some differences were observed in the infections using full-thickness tissues, primarily the extent of damage induced by the biofilms (Figs 6 and 7). Visually, very substantial damage was evident in the *in vitro* full-thickness tissues, with severe disruption of the epithelium (Fig. 6), whereas the relative extent of damage was reduced with MatTek full-thickness tissues. It was noted, however, that the epithelium of MatTek tissues, which by its very nature is an excellent natural physical barrier to the external environment, was more highly keratinised. Tissue invasion was observed in mixed-species infections of the MatTek tissues, although not to the same extent as seen in the *in vitro* tissues. Thus, although the same overall pattern of damage and invasion was evident in both *in vitro* and MatTek tissues, the duration of the infection with respect to MatTek tissues may not have been sufficient to demonstrate the pathogenicity of the infection that we have become accustomed to in previous studies. Furthermore, cornification of the tissue models may occur if, for example, they are extended long past the optimum culture period. This can subsequently influence tissue viability and responses to infection.

It is known that the pro-inflammatory cytokine interleukin-18 (IL-18) can stimulate monocyte cells to mature and induce phagocytosis of pathogens, and that monocyte cells can produce inflammatory cytokines of their own, including IL-23, the production of which during *in vitro* investigations was shown to be increased in a dose-dependent manner as a result of *C. albicans* challenge with prior stimulation by the bacterial LPS (Fig. S2). Furthermore, *IL-18* gene expression was up-regulated during mixed-species infection in this study and was also previously observed by Cavalcanti *et al.* [27].

Commercially available tissue models do not allow for the incorporation of immune cells within the tissues, but there is scope for continued development with *in vitro* full-thickness tissues to achieve this. The incorporation of immune cells may allow for cell-to-cell communication to be evaluated, along with the potential for the migration of immune cells to clear the infection to be monitored. This study is the first step in determining suitable models with which further development can be pursued. Neither the SkinEthic RHOE or MatTek EpiOral full-thickness tissues appear to provide benefits that outweigh the use or further development of *in vitro* tissue models, and there is a substantial cost benefit in using *in vitro* tissues, despite the increased manpower required to culture the *in vitro* tissues compared with simply purchasing them ready-made. There are clear advantages in using these for future studies instead of the commercially available tissues.

### Conclusions

The main findings of this study were the successful development of reproducible *in vitro* oral mucosal tissue models, and the observation that these in-house models were comparable to commercially available constructs for the study of denture-associated biofilm infections. This work highlights the flexibility, advantages and future potential of using *in vitro* tissues compared with static, commercially available constructs, and in particular the substantial cost benefit of doing so.

## Supplementary Data

191 Supplementary File 1
