# Supplementary material for: Denture-associated biofilm infection in three-dimensional oral mucosal tissue models
Source: J Med Microbiol. 2018 Jan 11;67(3):364–75. doi: 10.1099/jmm.0.000677 (PMC5882079; doi:10.1099/jmm.0.000677)
Supplement: Supplementary File 1 [file jmm-67-364-s001.pdf]

Fig. S1. Relative quantification of expression of the IL-18 gene in keratinocyte-only tissue model infections.

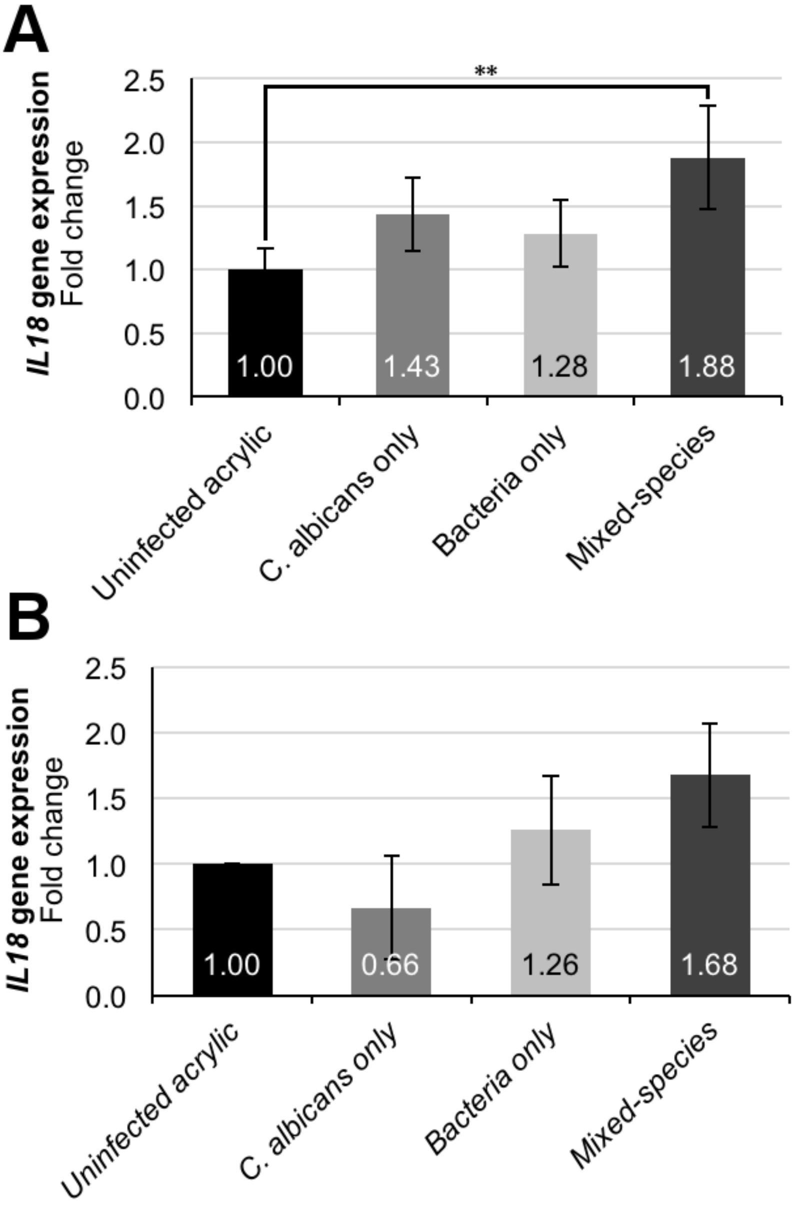

A similar pattern of expression was observed for both **(a)** SkinEthic™ RHOE and **(b)** *in vitro* keratinocyte-only tissues; a substantial increase in expression of IL-18 from tissues infected with mixed-species biofilms, but a lesser increase in the expression of the gene as a result of *C. albicans*-only or bacteria-only biofilms.

Fig. S2. Quantification of secreted IL-23 protein by THP-1 monocyte cells in response to lipopolysaccharide (LPS) and heat killed *Candida* (HKC) challenge.

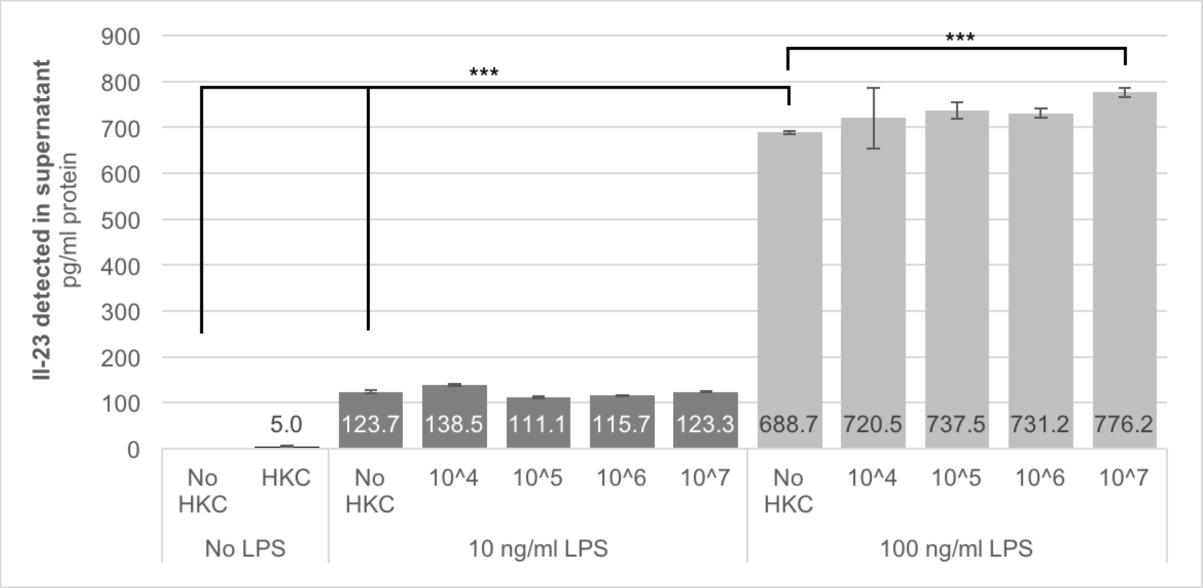

Observed statistically significant dose dependent increase in IL-23 when measured using ELISA correlated with both increasing stimulation with LPS, and subsequent increasing numbers of *Candida* cells.
